# The quality of research synthesis in surgery: the case of laparoscopic surgery for colorectal cancer

**DOI:** 10.1186/2046-4053-1-14

**Published:** 2012-02-17

**Authors:** Guillaume Martel, Suleena Duhaime, Jeffrey S Barkun, Robin P Boushey, Craig R Ramsay, Dean A Fergusson

**Affiliations:** 1 Department of Surgery, Department of Epidemiology & Community Medicine, and Ottawa Hospital Research Institute, The Ottawa Hospital, University of Ottawa, Ottawa, Ontario, Canada; 2 Department of Surgery and Division of Clinical Epidemiology, McGill University Health Centre, Montreal, Quebec, Canada; 3 Health Services Research Unit, Health Sciences Building, University of Aberdeen, Aberdeen, UK

**Keywords:** colorectal cancer, laparoscopy, surgery, systematic review, umbrella review

## Abstract

**Background:**

Several systematic reviews and meta-analyses populate the literature on the effectiveness of laparoscopic surgery for colorectal cancer. The utility of this body of work is unclear. The objective of this study was to synthesize all such systematic reviews in terms of clinical effectiveness, to appraise their quality, and to determine whether areas of duplication exist across reviews.

**Methods:**

Systematic reviews comparing laparoscopic and open surgery for colorectal cancer were identified using a comprehensive search protocol (1991 to 2008). The primary outcome was overall survival. The methodological quality of reviews was appraised using the Assessment of Multiple Systematic Reviews (AMSTAR) instrument. Abstraction and quality appraisal was carried out by two independent reviewers. Reviews were synthesized, and outcomes were compared qualitatively. A citation analysis was carried out using simple matrices to assess the comprehensiveness of each review.

**Results:**

In total, 27 reviews were included; 13 reviews included only randomized controlled trials. Rectal cancer was addressed exclusively by four reviews. There was significant overlap between review purposes, populations and, outcomes. The mean AMSTAR score (out of 11) was 5.8 (95% CI: 4.6 to 7.0). Overall survival was evaluated by ten reviews, none of which found a significant difference. Three reviews provided a selective meta-analysis of time-to-event data. Previously published systematic reviews were poorly and highly selectively referenced (mean citation ratio 0.16, 95% CI: 0.093 to 0.22). Previously published trials were not comprehensively identified and cited (mean citation ratio 0.56, 95% CI: 0.46 to 0.65).

**Conclusions:**

Numerous overlapping systematic reviews of laparoscopic and open surgery for colorectal cancer exist in the literature. Despite variable methods and quality, survival outcomes are congruent across reviews. A duplication of research efforts appears to exist in the literature. Further systematic reviews or meta-analyses are unlikely to be justified without specifying a significantly different research objective. This works lends support to the registration and updating of systematic reviews.

## Background

Any field of active investigation in healthcare requires that the overwhelming volume of cumulative information generated by individual researchers be condensed and summarized into a usable product. This synthesis must be simple, yet comprehensive, so as to inform decisions and policies carried out by physicians and surgeons, hospital administrators, healthcare payers, funding agencies, and other end users of research and outcomes data. Systematic reviews are widely considered to be the most comprehensive and unbiased method to do so [1]. On the basis of their completeness, such reviews should remain unique in the literature and be updated frequently, rather than duplicated or compartmentalized. In this context, some authors have recently advocated for the open registration of systematic reviews [2-4].

Laparoscopic colorectal surgery was first described in 1991 by Fowler and White [5] and by Jacobs and colleagues [6]. This technology has since been applied to almost every disease process, whether benign or malignant, involving the colon and rectum [7]. The use of laparoscopy rather than traditional open laparotomy to treat colorectal cancer has generated tremendous controversy in the surgical literature, particularly as concerns the oncologic adequacy of this technique. Many investigators have attempted to address this issue, and have thus generated a large body of literature over the past 20 years. Published studies have included the entire spectrum of research data, ranging from small personal case series to large nationally funded multicenter randomized controlled trials (RCTs). This work presents an excellent opportunity for a case study of research synthesis and knowledge translation processes in surgical research, an area that has traditionally lacked investigative rigor [8].

Numerous systematic reviews and meta-analyses pertaining to laparoscopic surgery for colorectal cancer have been published. Informal examination of these reviews would suggest significant overlap and possible duplication. The utility of this body of work is unclear at this time. As such, we set out to examine and appraise all existing systematic reviews of laparoscopic colorectal surgery for cancer, both in terms of clinical outcomes and their relative completeness, methodological quality, and overlap.

## Methods

This overview of systematic reviews was carried out using the framework for umbrella reviews described by the Cochrane Collaboration [9]. This approach consisted of identifying all existing systematic reviews and meta-analyses pertaining to laparoscopic surgery for colorectal cancer. This work was part of a larger review effort addressing both primary publications and review papers, the results of which will be presented separately. A review protocol was utilized for the project as a whole.

### Criteria for considering reviews for inclusion

All inclusion and exclusion criteria were defined *a priori*. All systematic reviews and meta-analyses addressing laparoscopic and open surgery for colorectal cancer were included. For this purpose, all reviews were allowable if they were self-described as systematic, whether in the title, abstract, or methods of the paper. Alternatively, a citation was also allowable if the authors presented a meta-analysis of primary papers or utilized meta-analytic techniques to pool primary data. These criteria were utilized regardless of the quality or comprehensiveness of the review. The type of primary data papers included in the citations could be RCTs, observational studies, or both.

All included citations reviewed primary papers addressing the curative resection of colon and/or rectal cancer. Patients with colorectal cancer did not have to be the sole population under review. Laparoscopic resection for colorectal cancer was the intervention under study. Included reviews had to present a comparison to an open resection control group.

The primary outcome of interest was overall survival. Secondary endpoints were also considered if they were included in a review of interest: operative outcomes, short-term postoperative outcomes, oncologic surrogate outcomes, long-term oncologic outcomes, other long-term outcomes, and quality of life. A review could be included in this overview even if no data pertaining to survival was presented, as identification of review deficits was a prespecified objective of our work. Reviews addressing exclusively cost or immune function were excluded.

### Search methods for identification of reviews

Systematic reviews and meta-analyses were identified as part of a broader comprehensive search strategy designed to identify primary comparative literature pertaining to laparoscopic and open surgery for colorectal cancer. The final search algorithm was devised in conjunction with an experienced information specialist from the Ottawa Hospital Library (Additional file 1). This search strategy was designed to be highly sensitive, and was modified from previously published work [10]. Six major databases were searched for relevant citations from 1991 to 2008 (Ovid MEDLINE, Ovid EMBASE, Cochrane Library, Science Citation Index Expanded, BIOSIS Preview, and BIREME LILACS). An additional 13 databases were also searched for relevant citations (Database of Abstracts of Reviews of Effectiveness, Heath Technology Assessment Database, NHS Economic Evaluation Database, NIHR Health Technology Assessment Programme, Trip Database, Clinicaltrials.gov, Controlled-trials.com, National Guidelines Clearinghouse, CMA Infobase: Clinical Practice Guidelines, NICE England, SIGN Scotland, NHMRC Australia, New Zealand Guidelines Group). The reference lists of all included citations were also screened to identify missing reviews. No language limitation was applied to the search strategy. All citation records were retrieved and downloaded electronically using Reference Manager 10 (ISI ResearchSoft, Berkeley, CA, USA), and were then de-duplicated.

### Selection of reviews

**Figure 1:**
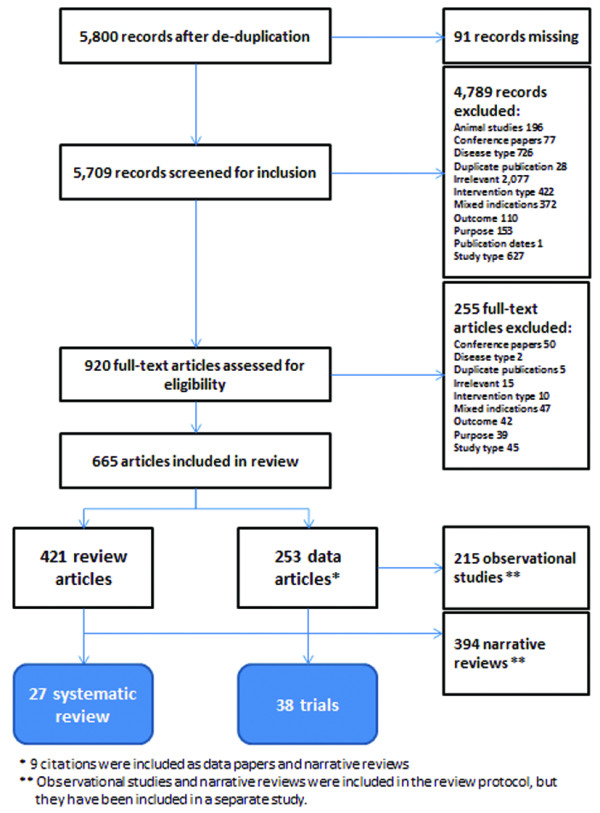
**PRISMA diagram **[95]. Note that the total number of data and review articles does not add up to 669, as 9 papers were included in both categories.

All citations were first screened for inclusion by one reviewer (GM) on the basis of titles and abstracts (Figure 1). All retained citations were then retrieved in full text. Papers that could not be obtained after extensive interlibrary searching were considered missing. Papers published in languages other than English, French, or Spanish were translated in full using Google Translate (Google Inc., Mountain View, CA, USA). Full-text articles were evaluated for inclusion by one reviewer (GM). Included articles were then classified as (1) data papers or (2) review papers. Review papers were then further divided as systematic reviews/meta-analyses, narrative reviews, textbook chapters, and guidelines/position papers. Only systematic reviews/meta-analyses were considered in the current work, while all other included paper types were set aside for a separate research project. All included systematic reviews were further evaluated for inclusion by a second reviewer (SD), and disagreements between the two reviewers was resolved by discussion and consensus.

### Data extraction and management

A total of 6 study characteristics and 19 prespecified outcomes of interests were extracted by two reviewers (GM, SD) onto data tables designed *a priori *(Table 1). All discrepancies were resolved by discussion and consensus. Relative outcomes were always recorded as a comparison of laparoscopic to open surgery. Where papers compared open to laparoscopic surgery, the appropriate reciprocal relative measure of effect and reciprocal measure of significance were calculated and recorded. The relative measures of effect were maintained in the statistical format provided by the authors of individual citations.

**Table 1:** List of characteristics and outcomes extracted from each review

Review characteristics	Review outcomes	
Date assessed as up to date	Positive radial margin (%)	Postoperative mortality (%, 30 day)
Population	Positive distal margin (%)	Length of hospital stay (days)
Intervention(s)	Radial margin distance (cm)	Overall morbidity (%)
Study types	Distal margin distance (cm)	Local recurrence (%)^a^
Reported outcomes	Operative time (min)	Distal metastases (%)^a^
Review limitations	Nodes harvested (n)^a^	Port site or wound metastases (%)^a^
	Anastomotic leak (%)	Cancer-related mortality (%)^a^
	Pneumonia (%)	Disease-free survival (%)^a^
	Urinary tract infection (%)	Overall survival (%)^a^
	Surgical site infection (%)	

^a^Oncologic outcomes included in Additional file 2.

### Assessment of methodological quality of included reviews

The methodological quality of individual reviews included in this overview was assessed using the Assessment of Multiple Systematic Reviews (AMSTAR) instrument [11]. This tool consists of 11 individual items, which were developed from pre-existing instruments, empirical evidence, and expert consensus. It has been shown to be valid, reliable, and relatively easy to use [11-13]. Each item within the instrument can receive 1 point, for a possible range of AMSTAR scores of 0 to 11. The AMSTAR instrument was administered independently by two reviewers (GM, SD) and discrepant scores were resolved by discussion and consensus.

### Data synthesis

The data extracted from each included systematic review was incorporated into summary tables and figures. The resulting data were synthesized in narrative form and correlated qualitatively with methodological AMSTAR scores. The congruence of pooled summary estimates for outcomes of interest across reviews was evaluated qualitatively.

As a means of evaluating the appropriateness of included reviews, a bibliographic analysis was carried out. Citation matrices [14] were generated that crosslinked individual reviews with (1) previously published systematic reviews identified in this work, and (2) published RCTs of laparoscopic vs open colorectal cancer surgery also identified in this work. This technique was used to determine whether the authors of included reviews made use of other pre-existing systematic reviews, and whether the identification of relevant RCTs was comprehensive. A 1-year lag time to publication was incorporated into the citation matrices and calculations. To account for the growing number of publications in time, the ratio of cited to total pre-existing publications was calculated for each systematic review. The mean ratios and 95% confidence interval were calculated for citation of both pre-existing systematic reviews and RCTs. Finally, citation ratios for individual systematic reviews were compared to AMSTAR scores and 5-year impact factors (where available) [15] using Pearson correlation coefficients.

All calculations and statistical analyses were performed using Microsoft Excel 2010 (Microsoft Corporation, Richmond, WA, USA) and SAS V. 9.2 (SAS Institute Inc., Cary, NC, USA).

## Results

### Description of included reviews

A total of 5,800 single citation records were screened for inclusion (Figure 1). After applying a 2-step screening process, a total of 27 full-text systematic reviews and/or meta-analyses [10,16-41] were included in this overview. Not included in this final tally was one Chinese language review, which could not be translated [42]. Similarly, another systematic review was excluded, as it focused solely on the methodological quality of RCTs identified in a Cochrane Review by the same author [43]. Finally, two more reviews were not considered as they addressed cost outcomes [44] and hand-assisted vs standard laparoscopic colorectal surgery [45], respectively. Other primary data papers and reviews identified in Figure 1 have also been included, and will form the basis for a separate publication. The full list of citations is available from the authors upon request.

The characteristics of all included reviews are provided in Table 2. Included reviews were published between 1994 and 2008, of which 19/27 (70%) were published in 2005 or later. Three groups published updates of their own reviews [10,17,19,23,29,39]. Because these were not labeled as updates in the titles or abstracts, they were included as independent reviews. A majority of reviews (n = 18, 67%) were published in traditional print journals, while the remainder comprised commissioned reports (n = 4, 15%) [16,17,23,24], Cochrane Reviews (n = 3, 11%) [26,30,38], and Health Technology Assessment (HTA) report (n = 2, 7.4%) [10,18]. The mean 5-year impact factor associated with published reports was 4.02 (n = 20, 95% CI: 3.07 to 4.96). These originated in 12 countries, with Europe (n = 14, 52%) and Australia (n = 5, 19%) accounting for a majority of papers. Five reviews were presented in non-English languages, including French (n = 2), Spanish (n = 2), and Norwegian (n = 1).

**Table 2:** Characteristics of included reviews

Review (origin)	Last update	Population/intervention^a^	Study types	Notes/limitations
Bernard/ANDEM, 1994 [16], (France)	July 1993	Any colorectal pathology and other abdominal pathologies (appendicitis, biliary disease, reflux, inguinal hernia)	Case series; no RCT or observational study found	In French. Paucity of available comparative literature. Broad review addressing all laparoscopic techniques (biliary, hernia, etc.).
Chapman/ASERNIP-S 2000 [17] (Australia)	July 1999	Colon cancer (right, left/sigmoid) and animal studies of *in vitro*/*in vivo *tumor spread. Excluded: transverse colectomy, APR, total colectomy.	RCTs, observational animal studies	Limited to English. Inclusion/exclusion of high and mid rectal cancer is not clearly specified. Chose not to pool data. Overlap with Chapman 2001 [19].
Vardulaki/NICE 2000 [18], (UK)	2000	Colorectal cancer. Excluded: case series of < 10 patients.	RCTs, observational	Extensive methodological description. Rigorous assessment of heterogeneity. Pooling of RCTs and observational data performed separately to avoid bias (for some outcomes). Statistical manipulations to overcome data limitations.
Chapman 2001 [19], (Australia)	July 1999	Colon cancer (right, left/sigmoid). Excluded: transverse colectomy, APR, total colectomy.	RCTs, observational	Limited to English. Inclusion/exclusion of high and mid rectal cancer is not clearly specified. Chose not to pool data. Overlap with Chapman 2000 [17].
Yong 2001 [20], (UK)	March 1997	Any colorectal pathology (all operation types)	Observational; no RCT found	Modification of inclusion/exclusion criteria from protocol based on available studies. Only 13/42 studies had only malignant disease. Pooled certain outcomes by calculating weighted means.
Korolija 2003 [21], (Croatia)	January 2000	'Colorectal procedures', unclear	RCTs, observational	No inclusion/exclusion criteria provided. Not mentioned whether RCTs included (two identified in reference list). Chose to pool outcomes from RCTs, observational studies, case series together (no mention of appropriateness).
Abraham 2004 [22], (Australia)	December 2002	Colorectal cancer (right hemicolectomy, left hemicolectomy, rectosigmoid resection, APR, others)	RCTs	Limited to English. Study selection on basis of reported outcomes.
Reza/UETS 2004 [23], (Spain)	September 2004	Colorectal cancer. Excluded: combination of benign and malignant pathologies, immune outcomes.	RCTs, systematic reviews, meta-analyses	In Spanish. Pre-2000 RCTs not included. No pooling of outcomes except survival and recurrence (reason unclear).
Poutignat/Haute Autorité de Santé 2005 [24], (France)	2003	Colorectal cancer. Excluded: metastatic disease, combined benign and malignant pathologies, non-intention-to-treat studies or those excluding converted patients.	RCTs, observational, meta-analysis	In French, limited to English and French. Unclear from text whether inclusion/exclusion criteria chosen *a priori *or after literature search by group of experts. Chose not to pool outcomes, qualitative analysis.
Manterola 2005 [25], (Chile)	December 2002	Colon cancer (right, transverse, left, sigmoid). Excluded: rectal cancer, perforated or obstructing cancer, metastasis, recurrent cancer, invasion into adjacent bowel/organs, polyps.	RCTs, observational	In Spanish, limited to English, Spanish, French, Italian. Methodology score created by review authors. Controlled series and RCTs broken down into individual case series yielding 6 laparoscopy and 11 open series in total.
Schwenk 2005 [26], (Germany)	January 2005	Colorectal pathologies (benign or malignant, for curative or palliative resection)	RCTs	Cochrane Review, protocol published in 2001. Extensive methodological description. Results for different pathologies pooled together.
Aziz 2006 [27], (UK)	2004	Rectal cancer (described as anterior resection or APR)	RCTs, observational	Extensive quantitative assessment of heterogeneity. Results for RCTs and observational studies pooled (no sensitivity analysis comparing RCT to observational data).
Tjandra 2006 [28], (Australia)	September 2005	Colon and rectosigmoid cancer. Excluded: rectal cancer, distant metastases.	RCTs	Limited to English. Effect of heterogeneity on results not clear/not documented.
Reza 2006 [29], (Spain)	November 2005	Colorectal cancer. Excluded: papers with mixed malignant/benign populations, immune function as outcome.	RCTs, systematic reviews	Pre-2000 RCTs not included. Limited description of methodology. No pooling of outcomes except survival and recurrence (reason unclear).
Breukink 2006 [30], (The Netherlands)	August 2006	Rectal cancer (undergoing total mesorectal excision)	RCTs, observational	Cochrane Review, protocol published in 2005. Primary outcome: disease-free survival. Chose not to pool survival data; qualitative analysis.
Gao 2006 [31], (China)	June 2005	Rectal cancer	RCTs, observational	Outcomes considered were selected *post hoc *after reviewing selected literature. Meta-analysis of RCT and observational data. Incomplete assessment of heterogeneity.
Murray 2006 [10], (UK)	May 2005	Colorectal cancer (including laparoscopic or HALS, excluding palliative surgery)	RCTs, IPD meta-analyses	HTA report (commissioned), protocol published in 2005. Pre-2000 RCTs identified from existing systematic reviews. Extensive description of methodology, rigorous assessment of heterogeneity. Academic-in-confidence data obtained from other authors removed from final report. Includes economic evaluation.
Kahnamoui 2007 [32], (Canada)	2004	Colorectal cancer (right, left, sigmoid, anterior resection, APR)	RCTs	Defined primary outcome: cancer-related mortality. Extensive methodological description. Quality appraisal list designed by authors.
Noel 2007 [33], (USA)	January 2005	Colorectal cancer, IBD, diverticular disease	RCTs, observational (controlled)	Limited to English. Excluded historical controls with < 50% overlap in accrual periods. Combination of RCT and non-RCT data. No assessment of methodological quality.
Bonjer 2007 [34], (The Netherlands)	2006	Colon cancer (rectal cancer included in at least one of trials)	RCTs	Minimum 150 patients with primary outcomes of survival. Authors of review are primary investigators in four included trials. Meta-analysis of individual patient data with 3 years of follow-up data.
Jackson 2007 [35], (USA)	February 2006	Colorectal cancer (colon and rectosigmoid as per inclusion criteria, but selected RCTs include rectal cancers). Excluded: benign pathologies.	RCTs	Primary outcome: survival and recurrence. Inclusion/exclusion of mid and low rectal cancer is not clearly specified. Meta-analysis of survival outcomes using time-to-event data. Significant assessment of heterogeneity.
Abraham 2007 [36], (Australia)	December 2003	Colorectal cancer (non-metastatic, treated with intention to cure). Excluded: uncontrolled series.	Observational	Limited to English. Limited to end of 2003. Quality assessment of papers performed but not utilized in analysis (to be reported separately). Limited assessment of heterogeneity.
Kuhry 2007 [37], (Norway)	April 2006	Colorectal cancer	RCTs	In Norwegian, limited to English. Very limited methodological description.
Kuhry 2008 [38], (Norway)	January 2008	Colorectal cancer (reporting long-term result, non-metastasized carcinoma)	RCTs	Cochrane review, protocol published 2002. Meta-analysis of survival outcomes using time-to-event data. Sensitivity analyses performed separately for colon and rectum.
Lourenco 2008 [39], (UK)	May 2007	Colorectal cancer. Excluded: patients undergoing palliative treatment.	RCTs, IPD meta-analyses	Pre-2000 RCTs identified from existing systematic reviews. Extensive description of methodology. Overlap with Murray 2006 [10].
Anderson 2008 [40], (USA)	November 2007	Rectal cancer. Excluded: tumors invading adjacent organs, previous pelvic surgery, contraindications to pneumoperitoneum, obstruction, perforation, studies which did not report rectal cancer outcomes separately.	RCTs, observational	Limited to English language. RCT and observational studies pooled together.
Liang 2008 [41], (China)	January 2007	Colorectal cancer. Excluded: emergency surgery (obstruction, perforation), known prohibitive adhesions, studies for which colorectal cancer patients could not be analyzed separately from patients with benign pathologies.	RCTs	Limited to English. Extensive assessment of heterogeneity.

^a^Laparoscopic versus open radical oncologic resection is the intervention under study unless stated otherwise.

APR = abdominoperineal resection; HALS = hand-assisted laparoscopic surgery; HTA = Health Technology Assessment; IBD = inflammatory bowel disease; IPD = individual patient data; RCT = randomized controlled trials.

Among included reviews, four (15%) addressed exclusively rectal cancer [27,30,31,41], one (3.7%) reviewed only colon cancer [25], whereas the remainder were less specific and identified 'colorectal cancer' as their population of choice (Tables 2 and 3). This descriptor led to contradictions in certain papers, as the authors sometimes specifically excluded rectal cancer, but then went on to include papers with 'rectosigmoid cancers' or 'anterior resections' [17,19,34]. A total of four (15%) reviews were wide in scope and addressed the laparoscopic treatment of all colorectal pathologies, of which colorectal cancer was a subset of patients [16,20,21,33]. All reviews sought to find studies comparing laparoscopic and open radical resection for cancer. One early paper found no controlled study, and was thus limited to case series of laparoscopic surgery for colorectal cancer [16]. Right hemicolectomy, left hemicolectomy, and sigmoid resection were the most commonly included types of colonic resections. All publications not limited exclusively to rectal cancer included those three procedures. In contrast, studies including transverse colectomies were frequently excluded by review authors [17,19,22,32].

**Table 3:** Summary of reviewed populations and outcomes

		Disease process			Outcomes reviewed					
		
Review	Purpose	Colon cancer	Rectal cancer	Other	Operative	Short-term postoperative	Oncologic surrogate	Long-term oncologic	Long-term other	QoL
Bernard 1994 [16]	Safety, efficacy	Y	Y	Y	Y	Y	Y	Y		
Chapman 2000 [17]	Safety, efficacy	Y	X		Y	Y	Y	Y		
Vardulaki 2000 [18]	Effectiveness, cost effectiveness	Y	Y		Y	Y	Y	Y		
Chapman 2001 [19]	Safety, efficacy	Y	X		Y	Y	Y	Y		
Yong 2001 [20]	Effectiveness	Y	Y	Y	Y	Y	Y	Y		
Korolija 2003 [21]	Extent of oncologic resection	Y	Y	Y			Y			
Abraham 2004 [22]	Safety, efficacy (short term)	Y	Y		Y	Y	Y			
Reza 2004 [23]	Safety, efficacy	Y	Y		Y	Y	Y	Y		Y
Poutignat 2005 [24]	Safety, efficacy	Y	Y		Y	Y	Y	Y		Y
Manterola 2005 [25]	Identify best therapeutic option	Y				Y		Y		
Schwenk 2005 [26]	Short-term benefits	Y	Y		Y	Y				Y
Aziz 2006 [27]	Short/long-term results (rectal)		Y		Y	Y	Y		Y	
Tjandra 2006 [28]	Update short-term results	Y	Y		Y	Y	Y			
Reza 2006 [29]	Safety, efficacy	Y	Y		Y	Y	Y	Y		
Breukink 2006 [30]	Safety, efficacy (rectal)		Y		Y	Y	Y	Y		Y
Gao 2006 [31]	Safety, efficacy, benefits (rectal)		Y		Y	Y	Y			
Murray 2006 [10]	Effectiveness, cost effectiveness	Y	Y		Y	Y	Y	Y	Y	Y
Kahnamoui 2007 [32]	Non-inferiority survival/perioperative results	Y	Y		Y	Y	Y	Y		
Noel 2007 [33]	Safety, efficacy (short term)	Y	Y	Y	Y	Y				
Bonjer 2007 [34]	Safety (oncologic)	Y	X				Y	Y		
Jackson 2007 [35]	Compare oncologic results	Y	Y				Y	Y		
Abraham 2007 [36]	Safety, efficacy (non-RCT, short term)	Y	Y		Y	Y	Y			
Kuhry 2007 [37]	Not stated	Y	Y		Y	Y	Y	Y		
Kuhry 2008 [38]	Evaluate long-term outcomes	Y	Y					Y	Y	
Lourenco 2008 [39]	Update, effectiveness	Y	Y		Y	Y	Y	Y	Y	Y
Anderson 2008 [40]	Compare oncologic outcomes (rectal)		Y				Y	Y		
Liang 2008 [41]	Evaluate recurrence outcomes	Y	Y					Y		

QoL = quality of life; × = Unclear from descriptions whether includes high rectal cancers.

All reviews except one set out to include RCTs as part of their analyses (Table 2). One group chose to focus solely upon observational studies [36]. A total of 13 (48%) reviews allowed only RCTs as part of their inclusion criteria. Of these, 77% were published in 2006 or later. One such group selected only four larger RCTs, and carried out a meta-analysis of individual patient data [34]. Two reviews whose last literature searches were in 1993 [16] and 1997 [20], respectively, were unable to identify any published RCTs. As a result, both groups presented only observational studies.

Table 3 provides an overview of the purposes, disease processes, and outcomes addressed by each systematic review. The self-described purposes were highly comparable, with most papers choosing to address issues of efficacy or effectiveness in broad terms. Long-term oncologic outcomes were sought by 19/27 reviewers (70%), while oncologic surrogates were found in 22 (81%) reviews. Similarly, operative and short-term postoperative outcomes were analyzed in 74% (n = 20) and 78% (n = 21) of reviews, respectively. A total of 13 (48%) reviews addressed all 4 outcomes types, while 18 (67%) included at least 3 of the 4. Table 3 reveals significant overlap in study purposes and outcomes. In contrast, long-term operative outcomes (n = 4, 15%) and perioperative quality of life (n = 6, 22%) were much less frequently included in the current group of systematic reviews.

### Methodological quality

**Figure 2:**
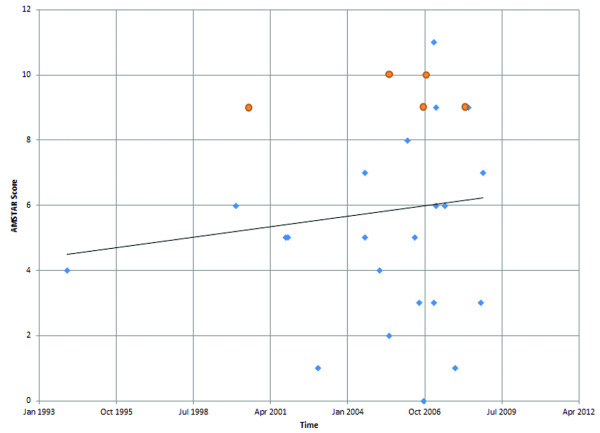
**Assessment of Multiple Systematic Reviews (AMSTAR) methodological quality scores against time**. Red circles represent Cochrane reviews and Health Technology Assessment (HTA) reports.

The included systematic reviews were generally of low to moderate quality. The mean AMSTAR methodological quality score was 5.8 (95% CI: 4.6 to 7.0). A total of eight reviews (30%) achieved a score of 9 or greater, and can be considered of high methodological quality. The quality of reviews appears to have improved modestly in time (Figure 2), with all but one high-quality review having been published in 2005 or later. Among high-quality reviews, three were published in traditional journals [32,35,39], three were Cochrane Reviews [26,30,38], and two were HTA reports [10,18].

**Figure 3:**
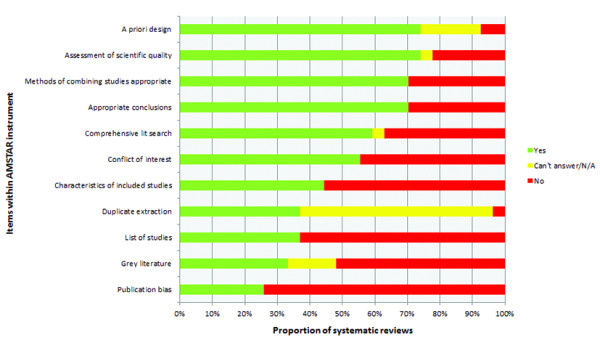
**Methodological quality of included reviews on the basis of individual Assessment of Multiple Systematic Reviews (AMSTAR) items**.

The composite of individual items within the AMSTAR score is presented in Figure 3. Most review groups defined their research question and inclusion criteria *a priori *(20/27, 74%), and documented the evaluation of the scientific quality of included studies (20/27, 74%). Similarly, 70% of systematic reviews used appropriate methods to combine individual study results (n = 19), and can be considered to have reached appropriate conclusions that reflected the methodological quality assessment of their selected papers (n = 19). In contrast, few groups addressed the possibility of publication bias (7/27, 26%), provided evidence of a duplicate study selection and data extraction process (10/27, 37%), listed all included and excluded studies (10/27, 37%), or avoided limiting their search or inclusion of studies on the basis of publication status (9/27, 33%).

### Synthesis of reviewed outcomes

**Figure 4:**
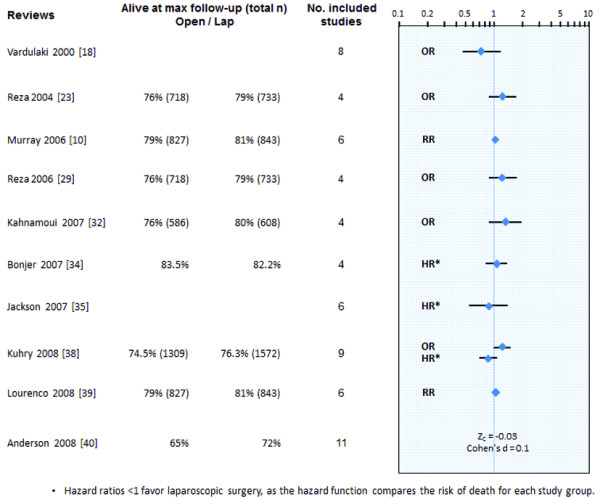
**Synthesis of overall survival across systematic reviews**.

The primary outcome of overall survival was pooled quantitatively in ten reviews (Figure 4 and Additional file 2). Most authors presented this data as a dichotomous outcome (alive/dead) at maximum follow-up. After meta-analysis, this data was presented as odds ratios (OR), risk ratios (RR), and Stouffer's composite Z (Z_C_). Two groups presented this outcome as time-to-event data by pooling hazard ratios (HR), using methods relying on the estimation of HR from Kaplan-Meier curves. Finally, another group also presented a HR that was derived from an individual patient data meta-analysis. No significant difference in overall survival was found between laparoscopic and open surgery for colorectal cancer across all meta-analytic comparisons. The direction of effect for all analyses favored laparoscopy, except for two which were conducted on observational studies and using a subset of studies with independent patient data [18,34].

All other pooled outcomes, except for the total number of harvested lymph nodes, yielded comparable non-significant results. These findings are not presented in the main text, but are available in Additional file 2.

### Bibliographic analysis

**Figure 5:**
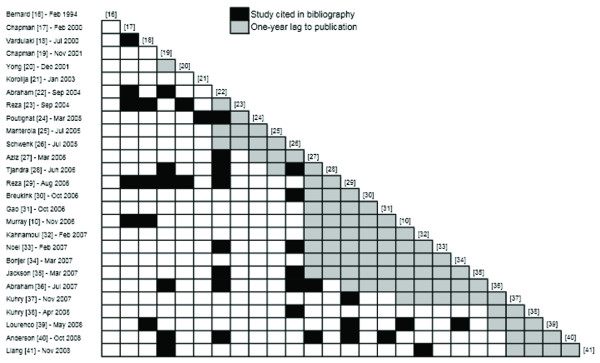
**Citation of previously published systematic reviews**.

The pattern of citations of previously published systematic reviews is presented in Figure 5. In total, nine (33%) reviews did not cite any pre-existing work. The maximum number of cited existing systematic reviews was five, which was found in two reviews [29,40]. All other studies cited zero to four pre-existing systematic reviews. The mean number of cited existing reviews was 1.6 (95% CI: 1.0 to 2.2). The mean ratio of cited to total existing systematic reviews was 0.16 (95% CI: 0.093 to 0.22), ranging from 0 to 0.5. All three Cochrane Reviews (0, 0.048 to 0.091) and the more recent HTA report (0.18) had citation ratios that were less than 0.2. There was no correlation between the citation ratio and the AMSTAR score (r = 0.047) or the journal's 5-year impact factor (r = -0.099) for individual reviews. In total, 13/26 pre-existing reviews were cited at least once, with 5 of these accounting for 71% of all citations (29/41) [17-19,22,26]. Figure 5 shows that five reviews were cited disproportionately more frequently, and that all five reviews were published in the earlier portion of the literature review.

**Figure 6:**
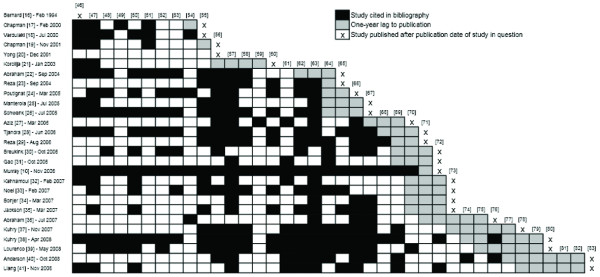
**Citation of previously published randomized controlled trials**.

The current overview protocol identified 38 publications pertaining to 23 individual RCTs comparing laparoscopic and open surgery for colorectal cancer [46-83]. The patterns of citations of these RCTs are presented in Figure 6. Two systematic reviews did not cite any RCTs. In the case of Bernard *et al*. [16], there were no RCTs yet published in the literature. In the case of Yong *et al*. [20], a total of ten RCT publications could have been cited. The mean ratio of cited to total published RCT reports was 0.45 (95% CI: 0.35 to 0.54), ranging from 0 to 1. Excluding Abraham *et al*. [36], which sought to analyze only observational studies, yielded a comparable mean ratio of 0.46 (95% CI: 0.36 to 0.56). Given that certain RCTs were published over multiple papers, the ratio of cited to total published RCT reports was recalculated using each trial as the denominator rather than individual papers. This analysis yielded a mean ratio of 0.56 (95% CI: 0.46, 0.65). A total of only four reviews identified at least 75% of RCT publications, of which two were Cochrane Reviews and one was an HTA report [10,26,28,38]. Reviews that selected rectal cancer as their sole patient population had generally low citation ratios, ranging from 0.13 to 0.39 [27,30,31,40]. Among all RCT publications, two were cited disproportionately more frequently than others. Indeed, the Barcelona trial by Lacy *et al*. [57] and the Clinical Outcomes of Surgical Therapy (COST) trial [63] were both cited by 90% of systematic reviews. Finally, the correlation between the citation ratios and the AMSTAR scores (r = 0.43), and between the ratios and the journal's 5-year impact factors were moderate at best (r = 0.46).

## Discussion

In this work, we have presented an overview of published systematic reviews and meta-analyses comparing laparoscopic and open surgery for colorectal cancer. We have conducted an extensive review of the literature using a highly sensitive search algorithm, and utilized the framework proposed by the Cochrane Collaboration [9] to synthesize 27 systematic reviews and meta-analyses published between 1994 and 2008. We have summarized the major oncologic outcome of overall survival, and provided a methodological appraisal of the reviews. Finally, we have analyzed the citation patterns of all included reviews in an attempt to understand the perceived redundancy of this body of work.

The first major finding of this overview pertains to the startling number of systematic reviews and meta-analyses identified in the literature on laparoscopic colorectal cancer surgery. There were more reviews than clinical trials, and 19 reviews were published within the span of 4 years (2005 to 2008). Given that systematic reviews are typically meant to be comprehensive in nature, this result is particularly surprising. This finding can be explained in part by the decision of certain reviewers to address only a subset of outcomes. That being said, the results shown in Table 3 would suggest that there is significant overlap between papers in terms of review purpose and outcomes. This argument is further supported by the large number of pooled analyses identified for the primary survival outcome in Figure 4 and in Additional file 2 in addition to the consistency and congruence of this result across reviews.

In addition to variation in outcomes, other authors have limited their review protocols with respect to disease subsets such as rectal cancer. This distinction may also account for a portion of the large volume of published systematic reviews, although it is unlikely to be a major factor as only four groups have focused solely on this population. More importantly, we argue that the limitation of review protocols to rectal cancer is artificial, as definitions and inclusion of rectal cancer in primary trials are highly variable. Indeed, high rectal lesions have been inconsistently defined both as rectosigmoid and rectal cancers, and this variation in terminology has been translated to different inclusion and exclusion criteria in systematic reviews. Given that high rectal cancers are typically treated in a manner that is more similar to sigmoid colon cancers, we argue that rectal cancer is best considered as part of broader reviews addressing colorectal cancer as a whole. Sensitivity analyses can then be carried out to identify outcome differences between colon and rectal cancer populations.

In addition to the above-mentioned patient population and outcomes factors, it is possible that the large number of systematic reviews be a result of a duplication of research efforts on the part of individual investigators. The analysis of citation patterns of pre-existing systematic reviews presented in Figure 5 supports this hypothesis. Indeed, we have identified that, on average, only 1.6 pre-existing reviews were referenced by individual systematic reviews. More strikingly, this corresponds to a mean ratio of cited to existing reviews of 0.16, indicating that, on average, 84% of existing systematic reviews in the literature were not found or ignored by individual review authors. This finding would support the argument that review authors either carry out poor literature evaluations or choose not to take into account pre-existing work in performing or analyzing their own reviews. Individual explanations are likely to vary, but it remains that our findings provide evidence of a duplication of research efforts in the literature.

Other examples of overlapping systematic reviews and meta-analyses can be found in the literature. In one such case, ten reviews pertaining to the use of *N*-acetylcysteine for the prevention of contrast-associated nephropathy were published within a 3-year span [84]. In that particular case study, the authors documented varying quality and inconsistent recommendations. Another such analysis was published in the field of orthopedic surgery, in which different methods of anterior cruciate ligament repair were compared [85]. A total of 11 overlapping systematic reviews were identified in this case, with varying conclusions. Interestingly, this group also identified evidence of incomplete citation of pre-existing systematic reviews.

Several academics have recently called for the registration of systematic reviews and their protocols [3,4]. Although the Cochrane Library currently acts as a central repository of high-quality systematic reviews and meta-analyses, it is clear from our analysis that most reviewers publish their work in traditional print journals. This interpretation is supported by Moher and colleagues, who reported that approximately 2,500 new systematic reviews are published annually, of which over 90% are found in specialty journals [2]. As such, a freely accessible registration system would have several advantages, including the early identification of pre-existing, ongoing, or unpublished reviews, the prioritization of research funding, and the enhancement of collaboration between review groups, while minimizing the possibility of research duplication. This body of information would be of particular use to practicing surgeons who would now have access to systematic reviews and meta-analyses through a single portal. Duplication of systematic reviews may be minimized with the existence of such an open-access registry, and identification of pre-existing work would likely be enhanced.

The continuous publication of new trials in time leads to the production and dissemination of new systematic reviews as a means to provide a synthesis of the literature that relies upon the latest data. For systematic reviews to be considered useful for end users, they must be up to date. In the current study, at least three groups of reviewers have provided updates of their own work in subsequent publications [10,17,19,23,29,39]. However, these were not specifically labeled as such, a finding that may lead to confusion on the part of the reader. In contrast, all three Cochrane Reviews documented having undergone substantive amendments since their original publication, as a result of new data pertinent data in the literature [26,30,38]. This difference between Cochrane and non-Cochrane reviews is not surprising. Indeed, Jadad *et al*. have previously demonstrated that only 3% of systematic reviews published in traditional journals underwent update within 2 years of publication, compared with 38% of Cochrane Reviews [86]. Although the timing at which systematic reviews should be updated remains controversial, it seems intuitive that, in a rapidly progressing field of healthcare such as laparoscopic surgery for colorectal cancer, existing systematic reviews should be updated frequently [87,88]. The case for updating systematic reviews becomes particularly compelling when one considers the large number of overlapping reviews identified in this study, and when registration of systematic reviews is considered.

The methodological quality of systematic reviews included in this study was low to moderate. As indicated, the mean AMSTAR score was 5.6, although 8/27 reviews achieved a score of ≥9. It is noteworthy that all Cochrane reviews and HTA reports in this study were classified as high-quality reviews. This result is supported by existing work in the literature, which demonstrated that Cochrane reviews have greater methodological rigor than traditional print reviews [86].

Although many systematic reviews in this work were deemed to have a comprehensive literature search (n = 16, 59%) on AMSTAR methodology scoring, few incorporated the gray literature (n = 9, 33%). As a result, many reports fell short in their identification of published trials. Indeed, the mean ratio of cited to existing RCT reports was only 0.46 across reviews. It was only marginally better at 0.56 if one considered actual trials rather individual reports of trials which were sometimes multiple (38 reports on 23 RCTs). Only one HTA report [10] managed to identify all reports of existing trials. This finding is concerning in that well conducted systematic reviews are meant to be comprehensive and commonly believed to provide the highest degree of healthcare evidence [4].

Many factors may explain the finding of incomplete citation, including the differing patient populations and outcomes examined in each review. This is particularly relevant given the recent report by the ORBIT group on outcome reporting bias and its potential impact upon results of systematic reviews [89]. As highlighted in the ORBIT study, it is highly important that systematic reviews avoid excluding trials on the basis of a lack of relevant data, as authors may have elected not to report a given outcome. In addition, it is also likely that the time between systematic review search completion and dissemination in electronic or print format may account for a portion of the poor citation of trials. We have attempted to avoid this form of time lag bias by allowing for a reasonable 1-year gray zone between citation of RCTs and publication (Figures 5 and 6). However, several systematic reviews had time lags to publication in excess of 2 years [19-21,25,32,36] (Table 2), which may partially account for a failure to identify more recent RCTs. In addition to the above factors, it is also likely that individual search strategies were not as comprehensive as should be, in order to identify all available trials. In the current study, we have modified the highly sensitive search algorithm developed by Murray and colleagues [10] and have identified at least two recent trials that have not been cited in of the existing reviews [71,73]. Moreover, at least four other reports of RCTs were cited much less frequently than other reports of trials published around the same time period [49,54,59,68,70]. In contrast, two well known RCTs were cited by almost all reviews [57,63]. Putting together the above patterns of trials citations, we argue that the identification of trial evidence was incomplete in most retrieved systematic reviews, due at least in part to inadequate search strategies.

Our overview of all systematic reviews presenting data on oncologic outcomes reveals several important findings. First, we found no evidence of a consistent or congruent difference in overall survival between patients with colon cancer treated by laparoscopy or open surgery. Similar conclusions can be drawn from other oncologic outcomes (data not shown; Additional file 2). This result is likely to be extendable to patients with high rectal cancer as many large trials included this pathology [62,66,76]. However, our analysis cannot be extrapolated to those with mid or low rectal cancer, as too few trials have included these patients. Results from the large multicenter and multinational COLOR II [90], ACOSOG Z6051 [91] trials will shed light onto this area of uncertainty. That being said, it is important to note that the above conclusion is limited by the lack of proper analysis of time-to-event data. Indeed, only three meta-analyses addressing overall survival presented their data in the form of HR [34,35,38]. Instead, many authors simply used pooled OR, which incorporate the proportion of patients alive or dead at a given point in time in each study. While this approach provides some information on survival, it is potentially biased by variable lengths of follow-up, different trial maturity, and the incomplete utilization of available data from patient censoring [38]. Because many RCT authors do not report HR, statistical methods exist to generate such estimates from Kaplan-Meier curves [92-94]. We advocate that review groups should attempt to gather this type of data when addressing survival or other oncologic outcomes.

## Conclusions

A large number of overlapping systematic reviews and meta-analyses comparing laparoscopic and open surgery for colorectal cancer can be identified in the literature. The methodological quality of systematic reviews is generally low to moderate, as evidenced by the incomplete identification of published trials. On the whole, Cochrane Reviews and Health Technology Assessment reports demonstrate higher quality indices than most traditional print reviews. Survival outcomes are inconsistently reported and time-to-event data are infrequently included in pooled estimates. That being said, all pooled estimates of overall survival comparing laparoscopic and open surgery for colorectal cancer are congruent and demonstrate no significant difference. There appears to be evidence of duplication of research efforts among review groups, as evidenced by overlapping review purposes, populations, and outcomes, as well as by the poor citation of pre-existing systematic reviews. Further systematic reviews or meta-analyses are unlikely to be justified without specifying a significantly different research objective. This works lends support to the registration and updating of systematic reviews.

## Competing interests

The Division of General Surgery and Department of Surgery at the Ottawa Hospital, University of Ottawa, are supported by unrestricted educational grants from Covidien Canada and Storz Canada.

## Authors' contributions

GM contributed to the conception and design of the study, provided clinical expertise, acquired, abstracted, analyzed, and interpreted the data, and drafted the manuscript. SD acquired, abstracted, analyzed, and interpreted the data. JSB contributed to the conception and design of the study, and revised the manuscript critically. RPB contributed to the conception and design of the study, provided clinical expertise, and revised the manuscript critically. CRR contributed to the conception and design of the study, analyzed and interpreted the data, and revised the manuscript critically. DAF contributed to the conception and design of the study, analyzed and interpreted the data, revised the manuscript critically, and provided global supervision of the project. All authors read and approved the final manuscript.

## Supplementary Material

Additional file 1 **Ovid MEDLINE (1950 to July Week 4 2008)**. Click here for file

Additional file 2 **Summary of oncologic outcomes**. Click here for file
