# Supplementary material for: The quality of research synthesis in surgery: the case of laparoscopic surgery for colorectal cancer
Source: Syst Rev. 2012 Feb 17;1:14. doi: 10.1186/2046-4053-1-14 (PMC3351744; doi:10.1186/2046-4053-1-14)
Supplement: Additional file 1 — Ovid MEDLINE (1950 to July Week 4 2008). [file 2046-4053-1-14-S1.DOC]

**Appendix 1**

**Ovid MEDLINE (1950 to July Week 4 2008)**

**Ovid MEDLINE In-Process & Other Non-Indexed Citations (4 August 2008)**

1. exp colorectal neoplasms/su

2. exp colectomy/

3. (colectom$ or hemicolect$ or colotom$).tw.

4. ((mesorect$ adj3 excision$) or (anterior adj3 resection) or abdominoperineal or proctectomy or proctocolectomy).tw.

5. or/1-4

6. exp colorectal neoplasms/

7. ((cancer or neoplas$ or adenocarcinoma$ or carcinoma$ or malignan$) adj3 (colorectal or colon$ or rectal or rectum or recto$ or sigmoid$ or instest$ or bowel)).tw.

8. or/6-7

9. adenocarcinoma/

10. carcinoma/

11. neoplasms/

12. or/9-11

13. exp colon/

14. rectum/

15. or/13-14

16. 12 and 15

17. colorectal surgery/

18. Surgical procedures,operative/

19. su.fs.

20. (surgery or surgical or surgeon$).tw.

21. resect$.tw.

22. operat$.tw.

23. or/17-22

24. (8 or 16) and 23

25. 5 or 24

26. laparoscopy/

27. Surgical procedures,minimally invasive/

28. (minimal$ adj3 (invasiv$ or access$)).tw.

29. laparoscop$.tw.

30. (key hole or keyhole).tw.

31. (hand assist$ or hand-assist$ or HALS or hand-port).tw.

32. or/26-31

33. 25 and 32

34. limit 33 to yr = 1991-2008

35. animal/not human/

36. 34 not 35

37. remove duplicates from 36

**Ovid EMBASE (1980 to 2008 Week 31)**

1. exp colon cancer/su

2. exp rectum cancer/su

3. exp colon resection/

4. exp rectum resection/

5. (colectom$ or hemicolect$ or colotom$).tw.

6. ((mesorect$ adj3 excision$) or (anterior adj3 resection) or abdominoperineal or proctectomy or proctocolectomy).tw.

7. or/1-6

8. exp colon cancer/

9. exp rectum cancer/

10. ((cancer or neoplas$ or adenocarcinoma$ or carcinoma$ or malignan$) adj3 (colorectal or colon$ or rectal or rectum or recto$ or sigmoid$ or instest$ or bowel)).tw.

11. or/8-10

12. adenocarcinoma/

13. carcinoma/

14. neoplasms/

15. or/12-14

16. exp colon/

17. exp rectum/

18. or/16-17

19. 15 and 18

20. colorectal surgery/

21. surgery/

22. su.fs.

23. (surgery or surgical or surgeon$).tw.

24. resect$.tw.

25. operat$.tw.

26. or/20-25

27. (11 or 19) and 26

28. 7 or 27

29. laparoscopy/

30. laparoscopic surgery/

31. Minimally invasive surgery/

32. (minimal$ adj3 (invasiv$ or access$)).tw.

33. laparoscop$.tw.

34. (key hole or keyhole).tw.

35. (hand assist$ or hand-assist$ or HALS or hand-port).tw.

36. or/29-35

37. 28 and 36

38. limit 37 to yr = 1991-2008

39. (animal/or nonhuman/) not human/

40. 38 not 39

41. remove duplicates from 40

**Cochrane Library (Issue 3, 2008)**

#1. MeSH descriptor Colorectal Neoplasms explode all trees with qualifier: SU

#2. MeSH descriptor Colectomy explode all trees

#3. colectom* in All Fields or hemicolect* in All Fields or colotom* in All Fields

#4. ((mesorect* NEAR/3 excision*) OR (anterior NEAR/3 resection) OR abdominoperineal OR proctectomy OR proctocolectomy) in All Fields

#5. (#1 OR #2 OR #3 OR #4)

#6. MeSH descriptor Colorectal Neoplasms explode all trees

#7. ((cancer OR neoplas* OR adenocarcinoma* OR carcinoma* OR malignan*) NEAR/3 (colorectal OR colon* OR rectal OR rectum OR recto* OR sigmoid* OR instest* OR bowel)) in All Fields

#8. (#6 OR #7)

#9. MeSH descriptor Adenocarcinoma, this term only

#10. MeSH descriptor Carcinoma, this term only

#11. MeSH descriptor Neoplasms, this term only

#12. (#9 OR #10 OR #11)

#13. MeSH descriptor Colon explode all trees

#14. MeSH descriptor Rectum, this term only

#15. (#13 OR #14)

#16. (#12 AND #15)

#17. MeSH descriptor Colorectal Surgery, this term only

#18. MeSH descriptor Surgical Procedures, Operative, this term only

#19. su.fs in All Fields

#20. (surgery OR surgical OR surgeon*) in All Fields

#21. (resect* OR operation*) in All Fields

#22. (#17 OR #18 OR #19 OR #20 OR #21)

#23. ((#8 OR #16) AND #22)

#24. (#5 OR #23)

#25. MeSH descriptor Laparoscopy, this term only

#26. MeSH descriptor Surgical Procedures, Minimally Invasive, this term only

#27. (minimal* NEAR/3 (invasiv* or access*)) in All Fields

#28. laparoscop* OR key hole OR keyhole OR hand assist* OR hand-assist* OR HALS OR hand-port in All Fields

#29. (#25 OR #26 OR #27 OR #28)

#30. (#24 AND #29)

**Science Citation Index Expanded (Web of Knowledge, 1991 to 14 August 2008)**

**BIOSIS Previews (Web of Knowledge, 1991 to 14 August 2008)**

#1. TS = (colectom* OR hemicolect* OR colotom*)

#2. TS = (mesorect* SAME excision*)

#3. TS = ((colon or colorectal) SAME resect*)

#4. #1 OR #2 OR #3

#5. TS = (cancer SAME (colorectal or colon* OR rectal OR rectum OR rectosigmoid OR cecal OR caecal OR intestin* OR bowel))

#6. TS = (carcinoma SAME (colorectal OR colon* OR rectal OR rectum OR intestin* OR rectosigmoid OR cecal OR caecal OR bowel))

#7. TS = (neoplas* SAME (colorectal OR colon* OR rectal OR rectum OR intestin* OR rectosigmoid OR cecal OR caecal OR bowel))

#8. TS = (adenocarcinoma* SAME (colorectal OR colon* OR rectal OR rectum OR intestin* OR rectosigmoid OR cecal OR caecal OR bowel))

#9. TS = (malignan* SAME (colorectal OR colon* OR rectal OR rectum OR intestin* OR rectosigmoid OR cecal OR caecal OR bowel))

#10. #5 OR #6 OR #7 OR #8 OR #9

#11. TS = laparoscop*

#12. TS = (minimal* SAME (invasiv* OR access*))

#13. TS = (key hole or keyhole)

#14. TS = (hand assist* OR hand-assist* OR HALS)

#15. #11 OR #12 OR #13 OR #14

#16. (#4 OR #10) AND #15

**BIREME LILACS (16 September 2008)**

(Colorectal or colon$ or rectal or rectum or recto$ sigmoid$) and (cancer or neoplas$ or adenocarcinoma$ or carcinoma$ or malignan$) and laparoscop$

**Database of Abstracts and Reviews of Effectiveness (http://crd.york.ac.uk, 16 September 2008)**

Colorectal AND laparoscop*

**Health Technology Assessment Database (http://crd.york.ac.uk, 16 September 2008)**

Colorectal AND laparoscop*

**NHS Economic Evaluation Database (http://crd.york.ac.uk, 16 September 2008)**

Colorectal AND laparoscop*

**NIHR Health Technology Assessment Programme (http://ncchta.org, 9 September 2008)**

‘Cancers’ ICD Chapter Heading, by ‘Surgery’

**TRIP Database (http://tripdatabase.com, 15 September 2008)**

Colorectal cancer AND laparoscopy

**Clinical Trials (http://clinicaltrials.gov, 15 September 2008)**

Colorectal AND laparoscopy

**Current Controlled Trials (http://controlled-trials.com, 15 September 2008)**

Colorectal AND laparoscop%

**National Guideline Clearinghouse (http://guidline.gov, 16 September 2008)**

Gastrointestinal Neoplasms MeSH Category

**CMA Infobase (http://www.mdm.ca/cpgsnew/cpgs/, 16 September 2008)**

Laparoscopy OR laparoscopic

**NICE England (http://nice.org.uk, 16 September 2008)**

(Colorectal OR colon OR rectal OR rectum) AND Surgical procedures

**SIGN Scotland (http://sign.ac.uk, 16 September 2008)**

Cancer topic

**NHMRC Australia (http://nhmrc.gov.au, 16 September 2008)**

Health guidelines

**New Zealand Guidelines Group (http://www.nzgg.org.nz, 16 September 2008)**

Cancer category, surgery category
