# Supplementary material for: The quality of research synthesis in surgery: the case of laparoscopic surgery for colorectal cancer
Source: Syst Rev. 2012 Feb 17;1:14. doi: 10.1186/2046-4053-1-14 (PMC3351744; doi:10.1186/2046-4053-1-14)
Supplement: Additional file 2 — Summary of oncologic outcomes. [file 2046-4053-1-14-S2.DOC]

**ADDITIONAL MATERIAL**

Summary of oncologic outcomes.

| **Review** | **Comparative Risk (%)** | | **Relative Effect***  **(95% CI)** | **P-value** | **No. patients Lap/Open or total (studies)** | **Notes** |
| --- | --- | --- | --- | --- | --- | --- |
|  | **OPEN** | **LAP** |  |  |  |  |
| ***Overall Survival*** | | | | | | |
| Vardulaki 2000 [18] |  |  | OR 0.74 (0.47, 1.16) | p=0.19 | (8) | All observational studies |
| Reza 2004 [23] | 545/718 (76%) | 577/733 (79%) | OR 1.23 (0.90, 1.72) | p=0.19 | 718/733(4) |  |
| Murray 2006 [10] | 655/827 (79%) | 684/843 (81%) | RR 1.03 (0.98, 1.09) | p=0.28 | 827/843(6) |  |
| Reza 2006 [29] | 545/718 (76%) | 577/733 (79%) | OR 1.23 (0.90, 1.72) | p=0.19 | 718/733 (4) |  |
| Kahnamoui 2007 [32] | 448/586 (76%) | 485/608 (80%) | OR 1.32 (0.90, 1.94) | p=0.15 | 586/608 (4) |  |
| Bonjer 2007 [34] | 83.5% | 82.2% | HR 1.07 (0.83,1.37) | p=0.61 | 1536(4) | IPD meta-analysis, Cox model for age, sex, stage |
| Jackson 2007 [35] |  |  | HR 0.88 (0.56, 1.39) | p=0.59 | 1577(6) | Time-to-event data |
| Kuhry 2008 [38] | 975/1309 (74.5%) | 1199/1572 (76.3%) | OR 1.19 (1, 1.43)  HR 0.89 (0.72, 1.08) | p=0.05 for OR | 2881 (9) for OR | Time-to-event data |
| Lourenco 2008 [39] | 655/827 (79%) | 684/843 (81%) | RR 1.03 (0.98, 1.09) | p=0.28 | 827/843(6) |  |
| Anderson 2008 [40] | 65% | 72% | Zc= -0.03, cohen’s d=0.1 | p=0.5 | 1995(11) |  |
| ***Disease-Free Survival*** | | | | | | |
| Vardulaki 2000 [18] |  |  | OR 0.60 (0.36, 0.98) | **p=0.043** | (5) | All observational studies |
| Murray 2006 [10] | 496/666 (74%) | 513/683 (75%) | RR 1.01 (0.95, 1.07) | p=0.83 | 666/683(4) |  |
| Bonjer 2007 [34] | 75.3% | 75.8% | HR 0.99 (0.80, 1.22) | p=0.92 | 1536(4) | IPD meta-analysis, Cox model for age, sex, stage |
| Lourenco 2008 [39] | 496/666 (74%) | 513/683 (75%) | RR 1.01 (0.95, 1.07) | p=0.83 | 666/683(4) | Heterogeneity (I^2^=59%) |
| ***Cancer-Related Mortality*** | | | | | | |
| Vardulaki 2000 [18] |  |  | OR 1.29 (0.86, 1.92) | p=0.219 | (8) | All Observational studies |
| Reza 2004 [23] | 47/290 (16%) | 41/298 (14%) | OR 0.70 (0.28, 1.72) | p=ns | 290/298(3) | Significant heterogeneity |
| Reza 2006 [29] | 47/290 (16%) | 41/298 (14%) | OR 0.70 (0.28, 1.72) | p=0.44 | 290/298(3) | Significant heterogeneity |
| Kahnamoui 2007 [32] | 91/586 (16%) | 67/608 (11%) | OR 0.67 (0.48, 0.94) | **p<0.05** | 586/608(5) |  |
| Jackson 2007 [35] |  |  | RR 0.80 (0.62, 1.04) | p=ns | 1577(6) |  |
| Kuhry 2008 [38] | 159/1108 (14.4%) | 185/1382(13.4%) | OR 0.84 (0.67, 1.06) | p=0.15 | 1108/1382(8) |  |
| ***Port-Site and/or Wound Metastases*** | | | | | | |
| Chapman 2000 [17] |  | 1.28% (0.64, 2.27) |  |  |  |  |
| Chapman 2001 [19] |  | 1.28% (0.64, 2.27) |  |  |  |  |
| Yong 2001 [20] |  | 0.8% |  |  |  |  |
| Reza 2004 [23] | 0-0.2% | 0-0.9% |  |  | (3) |  |
| Manterola 2005 [25] |  | 0.4% (0-0.9%) |  |  |  |  |
| Murray 2006 [10] | 1/637 (0.16%) | 2/649 (0.31%) | RR 1.97 (0.18, 21.62) | p=0.58 | 637/649(4) |  |
| Kahnamoui 2007 [32] | 0/149 (0%) | 1/159 (0.63%) | OR 1.36 (0.21, 8.80) | p=ns | 149/159(4) |  |
| Jackson 2007 [35] | 1/801 (0.12%) | 3/826 (0.36%) |  |  | 801/826(7) |  |
| Kuhry 2008 [38] | 5/1463 (0.34%) | 15/1724 (0.87%) | OR 1.97 (0.77, 5.02) | p=0.16 | 1463/1724(10) |  |
| Lourenco 2008 [39] | 1/485 (0.21%) | 5/997 (0.50%) |  |  | 485(3)/997(11) |  |
| Liang 2008 [41] | 2/356 (0.25%) | 2/342 (0.26%) | OR 1.04 (0.18, 6.03) | p=0.97 | 356/342(3) |  |
| ***Number of Harvested Lymph Nodes*** | | | | | | |
| Vardulaki 2000 [18] |  |  | WMD -0.34 (-1.34, 0.68) | p=0.516 | (6) | All observational studies |
| Korolija 2003 [21] | 10.7 | 11.8 | WMD 0.31 | p=0.558 |  |  |
| Abraham 2004 [22] |  |  | WR 0.98 | p=ns | 412(5) |  |
| Schwenk 2005 [26] |  |  | WMD 0.12 (-1.17, 1.41) | p=0.86 | 688 (7) | Colorectal cancer only |
| Aziz 2006 [27] |  |  | WMD -0.87 (-2.24,-0.49) | **p<0.001** | 925/ 550(17) | Rectal cancer only |
| Tjandra 2006 [28] | 12 | 12 | WMD 0.36 | p=0.48 | (9) |  |
| Murray 2006 [10] |  |  | WMD -0.41 (-1.42, 0.59) | p=0.42 | 453/458(3) |  |
| Bonjer 2007 [34] | 12.2 ± 7.8 | 11.8 ± 7.4 |  | p=0.40 | 1536(4) | Individual patient data |
| Jackson 2007 [35] |  |  | SMD -0.11 (-0.25, 0.04) | p=ns | 2671(9) |  |
| Abraham 2007 [36] |  |  | WR 0.98 | p=ns | 3063/ 1988(37) |  |
| Kuhry 2008 [38] |  |  | WMD -1.00 (-1.65, -0.35) | **p=0.0026** | 678/680(6) | Significant heterogeneity (I^2^=71%) |
| Lourenco 2008 [39] |  |  | WMD -0.41 (-1.42, 0.59) | p=0.42 | 911(3) |  |
| Anderson 2008 [40] | 11 | 10 | Zc= 3.0 | **p=0.001** | 2442(17) | Rectal cancer only |
| ***Local Disease Recurrence*** | | | | | | |
| Vardulaki 2000 [18] |  |  | OR 0.70 (0.38, 1.31) | p=0.262 | (9) | RCT and observational |
| Kahnamoui 2007 [32] | 19/187(10%) | 7/201 (3.5%) | OR 0.39 (0.17, 0.89) | **p=0.02** | 187/201(5) |  |
| Bonjer 2007 [34] | 40/740 (5.4%) | 29/796 (3.6%) |  | p=ns | 740/796(4) | Individual patient data |
| Kuhry 2008 [38] | 46/865 (5.3%) | 58/1122 (5.2%) | OR 0.81 (0.54, 1.22) | p=0.31 | 865/1122(8) |  |
| Anderson 2008 [40] | 8% | 7% | Zc= -0.2, Cohen’s d=0.1 | p=ns | 2277(16) |  |
| Liang 2008 [41] | 43/679 (6.3%) | 34/680 (5%) | OR 0.8 (0.5, 1.29) | p=0.36 | 679/680(7) |  |
| ***Distant Disease Recurrence*** | | | | | | |
| Vardulaki 2000 [18] |  |  | OR 1.05 (0.68, 1.61) | p=0.834 | (9) | RCT and observational |
| Bonjer 2007 [34] | 73/740 (9.9%) | 74/796 (9.3%) |  | p=ns | 740/796(4) | Individual patient data |
| Kuhry 2008 [38] | 100/796 (12.6%) | 140/1057 (13.2%) | OR 1.01 (0.76, 1.34) | p=0.93 | 796/1057(7) |  |
| Anderson 2008 [40] | 14% | 12% | Zc= -1, Cohen’s d=0.1 | p=0.54 | 1408(10) |  |
| Liang 2008 [41] | 71/584 (12.2%) | 65/579 (11.2%) | OR 0.90 (0.62, 1.29) | p=0.56 | 584/579(6) |  |
| ***Overall Disease Recurrence*** | | | | | | |
| Reza 2004 [23] | 143/718 (20%) | 132/733 (18%) | OR 0.89 (0.68, 1.16) | p=0.39 | 718/733(4) |  |
| Manterola 2005 [25] | 6.5% (0-10%) | 13.1% (0-21%) |  |  |  |  |
| Reza 2006 [29] | 143/718 (20%) | 132/733 (18%) | OR 0.88 (0.61, 1.27) | p=0.51 | 718/733(4) | I^2^=30% |
| Jackson 2007 [35] |  |  | RR 0.9 (0.73, 1.10)  HR 0.85 (0.53, 1.36) | p=ns  p=0.50 |  | Time-to-event data for HR |
| Lourenco 2008 [39] | 144/765 (19%) | 135/789 (17%) | RR 0.92 (0.74, 1.14) | p=0.44 | 789/765 (7) |  |
| Liang 2008 [41] | 190/1242 (15.3%) | 183/1232 (14.8%) | OR 0.93 (0.71, 1.21) | p=0.58 | 584/579(6) |  |

*All comparisons pertain to laparoscopic vs. open surgery; ratios compare laparoscopy/open surgery.

Abbreviations – HR: hazard ratio; OR: odds ratio; RR: risk ratio; SMD: standardized mean difference; WMD: weighted mean difference; WR: weighted ratio; Zc: Stouffer’s composite Zc.
